# Efficacy and safety of combination therapy with statin and ezetimibe in patients failing to achieve target LDL levels at cardiology outpatient clinics in Turkey (COM-TR-OLDL): a real-world observational study

**DOI:** 10.1186/s12944-026-02950-1

**Published:** 2026-05-06

**Authors:** Ayşe Çolak, Zeynep Kumral, Önder Öztürk, Ayşegül Ülgen Kunak, Ali Nizami Elmas, Ömer Bedir, Medeni Karaduman, Naci Babat, Umut Uyan, Nurcemal Şentürk, Sebahat Tekeli Şengül, Sefa Erdi Ömür, Şahbender Koç, Mehmet Ali Işık, Samet Uysal, Cennet Yıldız, Emre Asiltürk, Tuncay Güzel, Fahri Er, Alkım Ateşli Yazıcı, Emrah Yeşil, Fuat Bice, Cansu Öztürk, Murat Gökhan Yerlikaya, Lütfü Aşkın, Melisa Uçar, Murat Çakır, Ömer Uluuysal, Afag Özyıldız, Yusuf Demir Ozan, Tolga Kunak, Selçuk Öztürk, Özkan Karaca, Özkan Kayhan, Khayal Mirzayev, İlke Çelikkale, Emine Buket Kırdağ, Mehdi Zoghi, Asım Oktay Ergene

**Affiliations:** 1 Dokuz Eylul University Hospital, Izmir, Türkiye; 2 Unye State Hospital, Ordu, Türkiye; 3 Department of Cardiology, University of Health Sciences, Diyarbakır Gazi Yaşargil Training and Research Hospital, Diyarbakır, Türkiye; 4 Antalya Training and Research Hospital, Department of Cardiology, University of Health Sciences, Antalya, Türkiye; 5 İzmir Menderes State Hospital, İzmir, Türkiye; 6 Adana City Training and Research Hospital, Adana, Türkiye; 7 Van Training and Research Hospital, Van, Türkiye; 8 Van Yüzüncü Yıl University, Van, Türkiye; 9 Department of Cardiology, Ödemiş State Hospital, İzmir, Türkiye; 10 Faculty of Medicine, Department of Cardiology, Karadeniz Technical University, Trabzon, Türkiye; 11 Uşak University, Uşak, Türkiye; 12 Tokat Gaziosmanpaşa University, Tokat, Türkiye; 13 Department of Cardiology, Ankara Atatürk Sanatorium Training and Research Hospital, Ankara, Türkiye; 14 Mardin Training and Research Hospital, Mardin, Türkiye; 15 Şişli Memorial Hospital, Istanbul, Türkiye; 16 Bakırköy Dr. Sadi Konuk Training and Research Hospital, Istanbul, Türkiye; 17 Antalya Training and Research Hospital, Antalya, Türkiye; 18 Ağrı Training and Research Hospital, Ağrı, Türkiye; 19 Istanbul Bayrampaşa State Hospital, Istanbul, Türkiye; 20 Department of Cardiology, Mersin University, Faculty of Medicine, Mersin, Türkiye; 21 Niksar State Hospital, Tokat, Türkiye; 22 Trabzon Ahi Evren Thoracic and Cardiovascular Surgery Training and Research Hospital, Trabzon, Türkiye; 23 Gaziantep Islamic Science and Technology University, Gaziantep, Türkiye; 24 Samsun Training and Research Hospital, Samsun, Türkiye; 25 Erzincan Training and Research Hospital, Erzincan, Türkiye; 26 Yalova Training and Research Hospital, Yalova, Türkiye; 27 Recep Tayyip Erdoğan University, Training and Research Hospital, Rize, Türkiye; 28 Department of Cardiology, Fatsa State Hospital, Ordu, Türkiye; 29 Faculty of Medicine, Department of Cardiology, Akdeniz University, Antalya, Türkiye; 30 Faculty of Medicine, Department of Cardiology, Kırıkkale University, Kırıkkale, Türkiye; 31 Mersin City Training and Research Hospital, Mersin, Türkiye; 32 Kepez State Hospital, Antalya, Türkiye; 33 Koç University Hospital, Istanbul, Türkiye; 34 Department of Cardiology, Lokman Hekim University Hospital, Ankara, Türkiye; 35 Bafra State Hospital, Samsun, Türkiye; 36 Ege University Hospital, Izmir, Türkiye

**Keywords:** LDL-cholesterol, Atherosclerotic cardiovascular disease, Ezetimibe, Statins, Medication adherence, Fixed-dose combination, Dyslipidemia

## Abstract

**Background:**

Achieving target low-density lipoprotein cholesterol (LDL-C) levels in patients with atherosclerotic cardiovascular disease (ASCVD) frequently requires combination lipid-lowering therapy. Single-pill combination (SPC) regimens may improve adherence compared with free-dose combinations (FDC) in real-world clinical practice.

**Methods:**

This retrospective observational study included 450 ASCVD patients with baseline LDL-C levels of 70–189 mg/dL who were followed in cardiology outpatient clinics between January 2023 and December 2024. Patients received atorvastatin–ezetimibe either as a single-pill combination (SPC, *n* = 392) or as a free-dose combination (FDC, *n* = 58). Primary endpoints were LDL-C reduction and treatment adherence (≥ 80% of prescribed doses). Secondary endpoints included LDL-C target attainment and adverse events. Non-parametric tests and chi-square/Fisher’s exact tests were used for statistical analysis.

**Results:**

At 1 month, LDL-C levels were significantly lower in the SPC group compared with the FDC group (90.6 vs. 119 mg/dL; *p* = 0.005), and adherence was higher (89.8% vs. 70.7%; *p* < 0.001). At 4 months, LDL-C levels were comparable between groups, while continuous adherence remained significantly higher in the SPC group (86% vs. 69%; *p* = 0.001). A higher proportion of SPC-treated patients achieved LDL-C < 100 mg/dL at 1 month (81.9% vs. 67.2%; *p* = 0.009).

**Conclusions:**

In this real-world ASCVD cohort, SPC therapy with atorvastatin and ezetimibe was associated with superior adherence and comparable lipid-lowering efficacy despite lower statin doses. Fixed-dose combination strategies may represent an effective approach to optimize adherence and cardiovascular risk management.

## Background

Atherosclerotic cardiovascular diseases (ASCVD) represent a leading contributor to mortality worldwide. The data obtained from several different studies strongly and consistently demonstrate that low-density lipoprotein cholesterol (LDL-C) contributes directly to the pathogenesis of ASCVD [1], and it has been shown that a reduction of 39 mg/dL in LDL-C levels decreases mortality due to coronary artery disease by 20% [2]. Therefore, in individuals with established ASCVD, statin therapy at the highest tolerable dose, in conjunction with lifestyle modifications, is considered the front-line approach for lowering LDL-C values [3]. Nonetheless, achieving the recommended LDL-C targets with statin monotherapy alone is often challenging. As a result, combination therapies are frequently necessary [4].

Despite the demonstrated positive effects of lipid-lowering treatments in individuals with ASCVD, research has demonstrated that compliance with statin therapy is notably poor, with only 25% of patients maintaining regimen compliance at the 5-year follow-up [5]. In clinical practice, polypharmacy represents a critical factor that can adversely affect medication adherence among patients. In this regard, single-pill combination (SPC) therapies which integrates two or more drugs into a single pill, have been conclusively shown to significantly improve patient adherence, particularly in hypertensive individuals [6].

In lipid-lowering therapy, the SPC of statin and ezetimibe has been shown to significantly improve patient adherence compared to the group where these medications are prescribed as separate tablets [7]. However, since the data in this study were obtained from a healthcare system database, it is not known whether patients actually used the medication, and daily dosage data and lipid profile data are unavailable.

Hence, in this observational research, we sought to assess the impact of SPC therapy with atorvastatin and ezetimibe on lipid parameters, patient adherence, and the side effect profile, in comparison to patients who received statin and ezetimibe as separate prescriptions in a real-world setting.

## Methods

This observational study included subjects with established ASCVD and an LDL-C level ranging from 70 to 189 mg/dL, who referred to the cardiology outpatient service between January 2023 and December 2024 and were prescribed statin and ezetimibe either as free-dose combination (FDC) or in an SPC. In this study, the term single-pill combination (SPC) refers to a fixed-dose formulation containing atorvastatin and ezetimibe in a single tablet, whereas the term free-dose combination (FDC) refers to the prescription of atorvastatin and ezetimibe as separate tablets taken concomitantly.

Exclusion criteria encompassed subjects below 18 years, pregnant women, and those who were breastfeeding, those with active liver disease or persistent unexplained transaminase elevations (defined as ≥ 3 times the upper limit of normal), patients under immunosuppressive therapy (including cyclosporine, tacrolimus, or azathioprine), individuals receiving medications known to increase the risk of myopathy or rhabdomyolysis when combined with statins (such as itraconazole, ketoconazole, erythromycin, clarithromycin, telithromycin, or HIV protease inhibitors), patients with a history of alcohol abuse, individuals with known hypersensitivity or allergy to statins or ezetimibe, and those who declined or failed to provide written informed consent.

The clinical and demographic characteristics of the patients, along with the formulations and doses of statins prescribed, were retrospectively collected. Hypertension (HT) was defined as a systolic blood pressure (SBP) ≥ 140 mmHg, a diastolic blood pressure (DBP) ≥ 90 mmHg, or current use of antihypertensive therapy. Diabetes mellitus (DM) was diagnosed based on a fasting plasma glucose level ≥ 126 mg/dL or ongoing antidiabetic treatment. Chronic kidney disease (CKD) was defined as an estimated glomerular filtration rate (eGFR) below 60 mL/min/1.73 m².

Following inclusion, subjects were scheduled for follow-up evaluations at the 4th and 16th weeks. During the follow-up visits, the lipid profiles, medication adherence, and adverse events of the patients were recorded. Primary endpoints included the reduction in LDL-C levels and treatment adherence. Adherence was defined as taking ≥ 80% of the prescribed doses during the treatment period. Adherence data were collected based on patient self-reports during follow-up visits. Secondary endpoints were defined as the rates of patients reaching LDL-C thresholds of 100, 70, and 55 mg/dL, and the frequency of therapy-related adverse effects.

This study was conducted in accordance with the Declaration of Helsinki and approved by the Dokuz Eylül University Ethics Committee (protocol no: 615-SBKAEK). Written informed consent was obtained from all participants. Data are available from the corresponding author upon reasonable request in accordance with institutional regulations.

### Statistical analysis

All statistical analyses were performed using IBM SPSS Statistics for Mac (Version XX; IBM Corp., Armonk, NY, USA). Continuous variables are presented as median (minimum–maximum) and categorical variables as number (percentage). Between-group comparisons were conducted using the Mann–Whitney U test for continuous variables and the χ² test or Fisher exact test for categorical variables, as appropriate. Within-group comparisons between follow-up visits were performed using the Wilcoxon signed-rank test. A two-tailed p value < 0.05 was considered statistically significant. No post hoc multiple-comparison tests were applied.

## Results

### Patient characteristics

Baseline demographic and clinical features of the study cohort (*n* = 450) was outlined in Table 1. The median age of the patients were 60 years (interquartile range [IQR], 53–67 years), and 33% were female. HT and DM were present in 71% and 47% of patients, respectively. Active smoking was reported in 40% of the cohort. Prior occurrence of acute coronary syndrome (ACS) was present in 50% of patients, while 67% had undergone percutaneous coronary intervention (PCI), and 14% had a history of coronary artery bypass grafting (CABG). Noncritical coronary artery disease (CAD) was identified in 12% of the patients, and cerebrovascular disease/transient ischemic attack (CVD/TIA) was noted in 2.4%.

In this cohort, 85% of subjects were undergoing statin treatment without attaining target LDL-C concentrations, while 15% had discontinued statin therapy. The causes of statin discontinuation included adverse effects in 5.3% of patients, with myalgia being the most common (3.3%). Negative media reports were cited by 6.4% of patients, while other reasons accounted for 3.3% (Table 1).

**Table 1:** Baseline demographic and clinical data of all patients

Age (years)	All patients (*n* = 450)
	60 (53–67)
Female (n, %)	148 (32.9)
BMI (kg/m^2^)	27.7 (25.9–30.8)
SBP (mmHg)	130 (120–140)
DBP (mmHg)	80 (71–85)
HT (n, %)	321 (71.3)
DM (n, %)	212 (47.1)
Active smoker (n, %)	179 (39.8)
PAD (n, %)	15 (3.3)
CKD (n, %)	63 (14)
Prior ACS (n, %)	224 (49.8)
Prior PCI (n, %)	300 (66.7)
Prior CABG (n, %)	61 (13.6)
Presence of noncritical CAD (n, %)	55 (12.2)
CVD/TIA (n, %)	11 (2.4)
LVEF (%)	60 (50–60)
Patients not achieving LDL-C goals on statin therapy (n, %)	382 (84.9)
Discontinued statin therapy (n, %)	68 (15.1)
Side effect (n, %)	24 (5.3)
Myalgia (n, %)	15 (3.3)
Liver dysfunction (n, %)	1 (0.2)
CK elevation (n, %)	1 (0.2)
GIS symptoms (n, %)	4 (0.9)
Sleep disturbance (n, %)	1 (0.2)
Headache (n, %)	1 (0.2)
Erectile dysfunction	1 (0.2)
Negative media reports (n, %)	29 (6.4)
Other (n, %)	15 (3.3)
Non-compliance	12 (2.7)
Inability to access medication (n, %)	2 (0.4)
Absence of physician recommendation (n, %)	1 (0.2)

Abbreviations: *ACS* Acute coronary syndrome, *BMI* Body mass index, *CABG* Coronary artery bypass grafting, *CAD* Coronary artery disease, *CK* Creatine kinase, *CKD* Chronic kidney disease, *CVD* Cerebrovascular disease, *DBP* Diastolic blood pressure, *DM* Diabetes mellitus, *FDC* Fixed-dose combination, *GIS* Gastrointestinal system, *HT* Hypertension, *IQR* interquartile range, *LDL-C* Low-density lipoprotein cholesterol, *LVEF* Left ventricular ejection fraction, *PAH* Peripheral artery disease, *PCI* Percutaneous coronary intervention, *SBP* Systolic blood pressure, *TIA* Transient ischemic attack

### Baseline laboratory parameters

At baseline, the laboratory parameters were comparable between the FDC and the SPC group. No significant differences were observed in glucose, glycated hemoglobin (HbA1c), GFR, LDL-C, total cholesterol, triglycerides, aspartate aminotransferase (AST), alanine aminotransferase (ALT), or C-reactive protein (CRP) levels (Table 2). However, high-density lipoprotein cholesterol (HDL-C) and creatine kinase (CK) levels were significantly higher in the FDC group (47 vs. 43 mg/dL, *p* = 0.02, and 93 vs. 74 U/L, *p* = 0.015, respectively).

**Table 2:** Baseline laboratory data of the SPC and FDC groups

Glucose (mg/dL)	Single-pill combination (*n* = 392)	Free-dose combination (*n* = 58)	*P* value
	106.5 (94–139)	110 (91–144)	0.9
HbA1c (%)	6.1 (5.7–7.3)	6.2 (5.6–6.9)	0.88
GFR (mL/min/1.73 m^2^)	81 (69–94)	87 (70–96)	0.48
LDL-C (mg/dL)	134.8 (112–154)	143 (117–163)	0.17
HDL-C (mg/dL)	43 (38–51)	47 (41–55)	**0.02**
Total cholesterol (mg/dL)	211 (187–242)	217 (194–242)	0.32
Triglycerides (mg/dL)	178 (122–264)	175 (115–253)	0.85
AST (U/L)	21 (16–26)	21 (18–27)	0.27
ALT (U/L)	21 (15–28)	22 (16–31)	0.27
CK (U/L)	74 (47–100)	93 (53–127)	**0.015**
CRP (mg/L)	3 (2–5.6)	2.6 (1.7–4.3)	0.42

Abbreviations: *ALT* Alanine aminotransferase, *AST* Aspartate aminotransferase, *CK* Creatine kinase, *CRP* C-reactive protein, *GFR* Glomerular filtration rate, *HbA1c* Glycated hemoglobin, *HDL-C* High-density lipoprotein cholesterol, *LDL-C* Low-density lipoprotein cholesterol

### Initial treatment and assigned regimens

The initial statin therapy before inclusion for both groups are represented in Table 3. Prior to inclusion, atorvastatin 40 mg was the most common prescribed regimen in the SPC group (44.6%), whereas atorvastatin 80 mg (25.9%), rosuvastatin 20 mg (13.8%), and rosuvastatin 40 mg (13.8%) were utilized more often in the FDC group. Overall, patients in the FDC group tended to receive higher-intensity statins compared to those in the SPC group.

**Table 3:** Initial lipid-lowering treatment regiments prior to inclusion

	Single-pill combination (*n* = 392)	Free-dose combination (*n* = 58)
Initial treatment (*n*, %)		
Atorvastatin 10 mg	22 (5.6)	1 (1.7)
Atorvastatin 20 mg	97 (24.7)	7 (12.1)
Atorvastatin 40 mg	175 (44.6)	6 (10.3)
Atorvastatin 80 mg	19 (4.8)	15 (25.9)
Rosuvastatin 10 mg	9 (2.3)	6 (10.3)
Rosuvastatin 20 mg	30 (7.7)	8 (13.8)
Rosuvastatin 40 mg	15 (3.8)	8 (13.8)
Pitavastatin 2 mg	14 (3.6)	4 (6.9)
Pitavastatin 4 mg	11 (2.8)	3 (5.2)
Pravastatin 40 mg	0 (0)	1(1.7)

The assigned regimens after inclusion for both groups are represented in Table 4. Atorvastatin 40 mg + ezetimibe 10 mg was the main regimen in the SPC group (74.7%), whereas a broader distribution of high-intensity combinations—particularly atorvastatin 80 mg + ezetimibe and rosuvastatin-based combinations—was observed in the FDC group. Specifically, 84% of patients in the SPC group received a high-intensity statin plus ezetimibe regimen. Additionally, the mean atorvastatin dose in the SPC group was 34 mg, while the mean atorvastatin dose in the FDC group was 61 mg.

**Table 4:** Assigned treatment regimens at the time of inclusion

	Single-pill combination (*n* = 392)	Free-dose combination (*n* = 58)
Assigned treatment (*n*, %)		
Atorvastatin 10 mg + Ezetimibe 10 mg	24 (6.1)	0 (0)
Atorvastatin 20 mg + Ezetimibe 10 mg	75 (19.1)	4 (6.9)
Atorvastatin 40 mg + Ezetimibe 10 mg	293 (74.7)	9 (15.5)
Atorvastatin 80 mg + Ezetimibe 10 mg	0 (0)	19 (32.8)
Rosuvastatin 10 mg + Ezetimibe 10 mg	0 (0)	3 (5.2)
Rosuvastatin 20 mg + Ezetimibe 10 mg	0 (0)	9 (15.5)
Rosuvastatin 40 mg + Ezetimibe 10 mg	0 (0)	12 (20.7)
Pitavastatin 4 mg + Ezetimibe 10 mg	0 (0)	1 (0.2)
Pravastatin 40 mg + Ezetimibe 10 mg	0 (0)	1 (0.2)

### Primary endpoints: LDL-C Reduction and Treatment Adherence

**Fig. 1:**
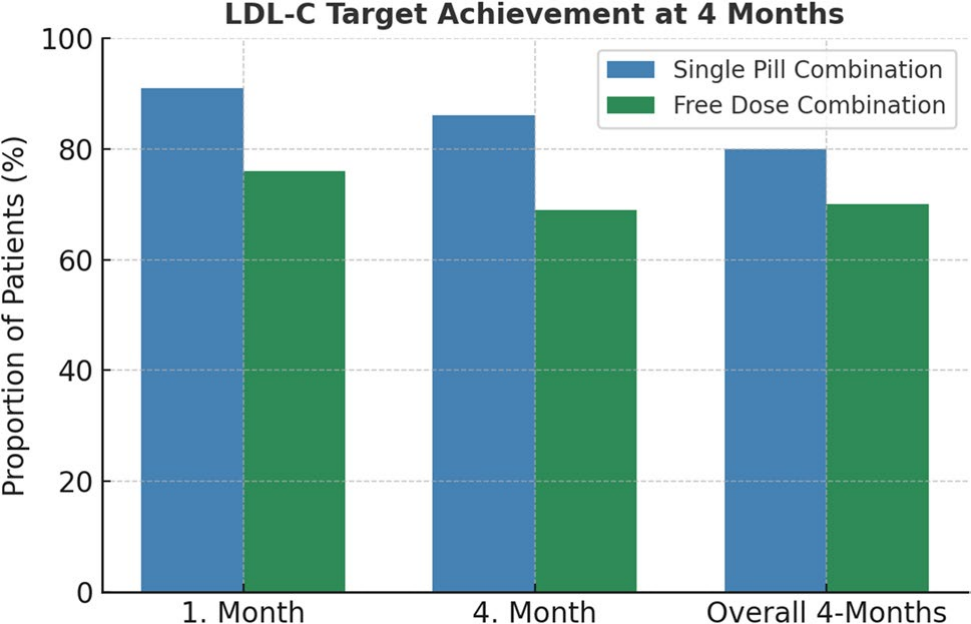
SPC vs. FDC LDL-C target achievement at 4 months

LDL-C reduction and treatment adherence outcomes in the FDC and SPC groups was outlined in Table 5. At the first month of follow-up, LDL-C levels were significantly lower in the SPC group compared to the FDC group (median: 90.6 mg/dL [IQR: 71–119] vs. 119 mg/dL [IQR: 80–135]; *p* = 0.005). Although LDL-C levels remained lower at the fourth month in the SPC group, the difference was not statistically significant (72 mg/dL [58–93] vs. 81 mg/dL [59–104]; *p* = 0.09). However, this observation should be interpreted with caution in light of the limited statistical power, and a type II error cannot be excluded. Similarly, the percentage reduction in LDL-C at the fourth month did not differ significantly between the groups (44% [32–53] vs. 41% [27–53]; *p* = 0.25) (Fig. 1).

**Table 5:** LDL-C reduction and treatment adherence in the FDC and SPC groups

Outcome	Single-pill combination(n=392)	Free-dosecombination(n=58)	*P *value
LDL-C at 1st month (mg/dL)	90.6 (71 – 119)	119 (80 – 135)	0.005
LDL-C at 4th month (mg/dL)	72 (58 – 93)	81 (59 – 104)	0.09
LDL-C reduction at 4th month (%)	44 (32 – 53)	41 (27 – 53)	0.25
Adherence at 1st month (n, %)	352 (89.8)	41 (70.7)	<0.001
Adherence at 4th month (n, %)	372 (94.9)	56 (96.6)	0.83
Adherence throughout 4 months (n, %)	337 (86)	40 (69)	0.001

**Fig. 2:**
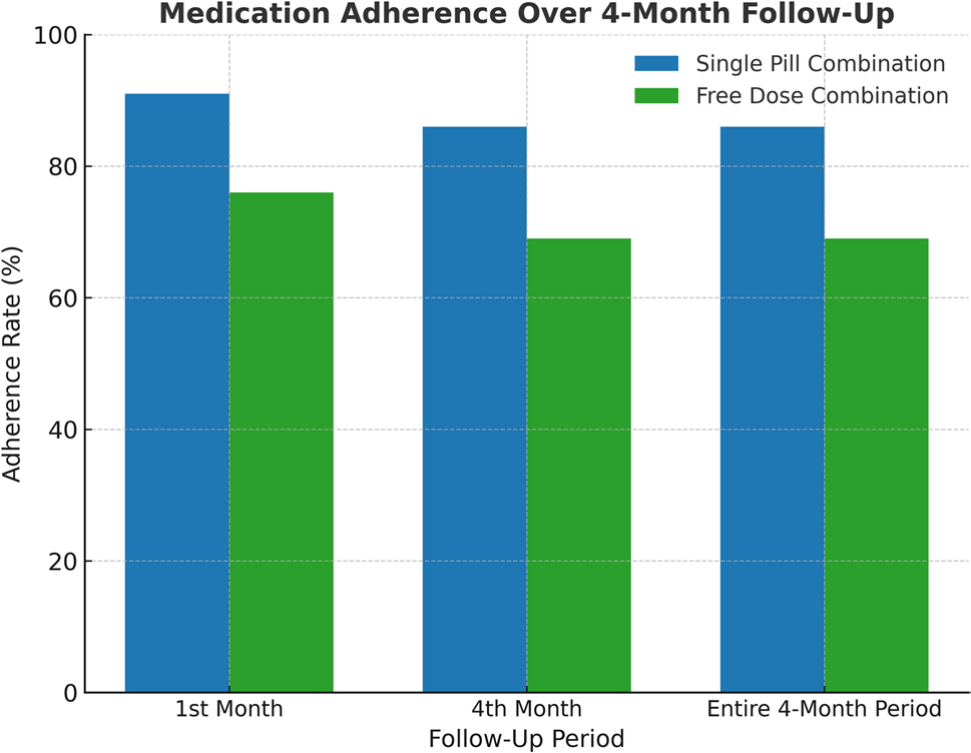
SPC vs. FDC medication adherence over 4-month follow-up

Adherence at the first month was significantly higher in the SPC group (89.8% vs. 70.7%; *p* < 0.001), whereas adherence rates at the fourth month were similar between groups (94.9% vs. 96.6%; *p* = 0.83). Importantly, continuous adherence throughout the 4-month follow-up period was significantly better in the SPC group (86% vs. 69%; *p* = 0.001) (Fig. 2).

### Secondary endpoints: LDL-C threshold achievement and treatment-related side effects

The secondary endpoints, including LDL-C threshold achievement and treatment-related adverse events at the 1st and 4th months of follow-up was represented in Table 6.

At the 1st follow-up, a significantly higher proportion of patients in the SPC group achieved the LDL-C target of < 100 mg/dL compared to the FDC group (81.9% vs. 67.2%, *p* = 0.009). No significant intergroup differences were observed for achieving lower LDL-C targets of less than 70 mg/dL and 55 mg/dL. In terms of safety, adverse event rates were generally low in across groups. Myalgia occurred slightly more often in the FDC group at both the first and fourth months, though not statistically significant. Notably, CK elevation < 10×ULN at the fourth month was significantly more prevalent in the FDC group (15.5% vs. 7.7%, *p* = 0.047), as was mild AST/ALT elevation < 3×ULN (6.9% vs. 1.3%, *p* = 0.02). No cases of rhabdomyolysis were reported.

**Table 6:** LDL-C cut-off achievement rates and frequency of treatment-related adverse events

LDL-C ≤ 100 mg/dL (*n*, %)	Single-pill combination (*n* = 392)	Free-dose combination (*n* = 58)	*P*-value
	321 (81.9)	39 (67.2)	0.009
LDL-C ≤ 70 mg/dL (n, %)	186 (47.4)	22 (37.9)	0.17
LDL-C ≤ 55 mg/dL (%)	87 (22.2)	10 (17.2)	0.39
Adverse events at 1st month (n,%)			
Myalgia	10 (2.6)	4 (6.9)	0.07
Rhabdomyolysis	0 (0)	0 (0)	NA
CK elevation < 10xULN	39 (9.9)	6 (10.3)	0.9
AST/ALT elevation <3xULN	11 (2.8)	3 (5.2)	0.3
AST/ALT elevation >3xULN	2 (0.5)	0 (0)	NA
Other adverse events			
GI symptoms	3 (0.8)	0 (0)	NA
Dermatitis	0 (0)	1 (1.7)	NA
Adverse events at 4th month (n, %)			
Myalgia	13 (3.3)	3 (5.2)	0.47
Rhabdomyolysis	1 (0.3)	0 (0)	NA
CK elevation < 10xULN	30 (7.7)	9 (15.5)	**0.047**
AST/ALT elevation <3xULN	5 (1.3)	4 (6.9)	**0.02**

Abbreviations: *LDL-C* Low-density lipoprotein cholesterol, *CK *Creatine kinase, *ULN* Upper limit of normal, *AST* Aspartate aminotransferase, *ALT* Alanine aminotransferase, *GI* Gastrointestinal

## Discussion

Our study showed that SPC with atorvastatin and ezetimibe treatment was associated with greater treatment adherence compared with the FDC therapy group. While the SPC group exhibited a more pronounced LDL-C reduction at the first-month follow-up, LDL-C levels at the fourth month were comparable between the FDC and SPC groups, although this finding should be interpreted with caution given the limited statistical power. Furthermore, despite the administration of relatively higher statin doses in the FDC group, the proportion of patients achieving LDL-C levels below 100 mg/dL was notably higher in the SPC group. Despite the low overall incidence of adverse events in both groups, CK elevations and mild increases in liver enzymes were more commonly observed in the FDC group than in the SPC group.

Maintaining adherence to statin therapy is of paramount importance in both primary and secondary ASCVD prevention. Suboptimal statin adherence in the first-year post-acute myocardial infarction was linked to a 1.7-fold rise in all-cause mortality and a 1.6-fold increase in CV mortality in the following year, emphasizing the significance of continuous statin therapy for favorable long-term outcomes [8]. Among those aged 75 years and above, stopping statins corresponded to a markedly increased incidence of major adverse CV events —1.3-fold in primary and 1.25-fold in secondary prevention—underscoring the clinical relevance of sustained statin use [9]. Treatment adherence is influenced by a complex interplay of factors pertaining to the patient, the pathology, the clinician, the treatment, and the broader healthcare infrastructure. Among therapy-related strategies, the implementation of polypill approaches has been strongly advocated to enhance adherence and improve long-term clinical outcomes [10]. Our results are consistent with prior large-scale observational studies investigating the combination of statin and ezetimibe administered either as a FDC or a SPC, which have consistently demonstrated superior adherence associated with SPC regimens. One such study reported significantly higher adherence rates and a 17% reduction in major adverse CV events in patients receiving SPC therapy over a median follow-up of 4.7 years [7]. Although the follow-up duration in our study was relatively shorter, we similarly observed significantly better adherence in the SPC group during the 4-month period.

Another key factor contributing to non-adherence in statin therapy is statin intolerance, defined as the inability to tolerate the statin dose necessary to achieve an adequate reduction in an individual’s CV risk [11]. Although true statin intolerance is relatively uncommon, perceived intolerance—often driven by statin-associated muscle symptoms—remains highly prevalent in clinical practice and contributes to treatment discontinuation. Contemporary evidence supports a patient-centered management approach, including dose reduction, switching to alternative statins, or implementing alternate dosing regimens. Importantly, the early addition of non-statin therapies such as ezetimibe represents an effective strategy to maintain lipid control while minimizing statin exposure and improving tolerability [12]. While statin intolerance may not influence overall mortality, it has been linked to substantially elevated cardiovascular risk, with observational data indicating a 36% higher likelihood of recurrent myocardial infarction and a 43% increase in ASCVD events [13]. Among the various risk factors associated with statin intolerance, high-intensity statin therapy has been demonstrated to be a key factor [11]. Given that high-intensity statin therapy is a well-recognized risk factor for statin intolerance, our findings are particularly noteworthy: despite receiving significantly lower atorvastatin doses (mean: 34 mg vs. 61 mg), patients in the SPC group achieved LDL-C reductions in line with those observed in the FDC group, in whom high-dose statins were more frequently prescribed. The superior treatment adherence observed in the SPC group throughout the study period likely contributed to more consistent LDL-C reduction. This may also suggest a potential synergistic lipid-lowering effect of the SPC of atorvastatin and ezetimibe, which may offer an effective and practical strategy for minimizing the risk of intolerance while maintaining therapeutic efficacy in real-world clinical practice.

Beyond its lipid-lowering efficacy, the addition of ezetimibe to statin therapy has been shown to provide incremental cardiovascular benefit. The IMPROVE-IT trial demonstrated that ezetimibe added to statin therapy significantly reduced major adverse cardiovascular events compared with statin monotherapy, establishing the clinical relevance of combination therapy beyond LDL-C reduction alone. More recent evidence further supports the role of combination therapy in high-risk populations, reinforcing guideline recommendations that advocate early intensification of lipid-lowering treatment when LDL-C targets are not achieved. These findings provide an important clinical framework for interpreting our results, where improved adherence with SPC therapy may further enhance the long-term cardiovascular benefit of combination treatment [14].

In addition to absolute LDL-C reduction, emerging evidence suggests that visit-to-visit variability in LDL-C levels may represent an independent determinant of cardiovascular risk. Recent studies have demonstrated that more stable lipid control is associated with improved cardiovascular outcomes, including a recent analysis evaluating LDL-C variability in patients treated with PCSK9 inhibitors and inclisiran [15]. In this context, therapies that improve adherence—such as single-pill combinations—may contribute not only to greater LDL-C reduction but also to reduced variability in lipid levels over time. This concept provides an additional mechanistic explanation for the potential long-term benefits of improved adherence observed in our study. This may be particularly relevant in our study population, where improved adherence observed with SPC therapy could contribute to more stable lipid profiles over time.

From a pharmacological perspective, the combination of atorvastatin and ezetimibe targets complementary pathways of cholesterol metabolism by inhibiting hepatic cholesterol synthesis and intestinal cholesterol absorption, respectively. This dual mechanism may allow effective LDL-C reduction at lower statin doses, potentially mitigating dose-related adverse effects while preserving lipid-lowering efficacy. In our study, patients receiving SPC therapy achieved LDL-C reductions comparable to those in the FDC group despite significantly lower statin intensity, supporting the concept of pharmacological synergy and improved tolerability associated with fixed-dose combination strategies.

Our study has several notable strengths. It reflects real-world clinical practice and includes a well-characterized cohort of patients with established ASCVD. Both treatment adherence and lipid-lowering efficacy were evaluated at two time points, and detailed information on baseline characteristics, treatment regimens, and adverse events was provided. Importantly, a post hoc power analysis was conducted, demonstrating adequate power for adherence outcomes, thus supporting the reliability of this finding.

However, several limitations should be acknowledged. The retrospective design limits causal inference and may introduce bias. The smaller sample size in the FDC group may have reduced the power to detect differences in LDL-C reduction; indeed, the power for this outcome was only 38.1% (Cohen’s d = 0.23). Importantly, the difference in baseline statin intensity between the SPC and FDC groups represents a major potential confounder, as higher statin doses in the FDC group may have independently influenced LDL-C reduction. Therefore, the observed lipid-lowering effects cannot be attributed solely to the treatment formulation. Given the limited statistical power for LDL-C reduction, the absence of a significant difference between groups at 4 months should be interpreted with caution, and the possibility of a type II error cannot be excluded. The retrospective design and limited sample size precluded comprehensive multivariable adjustment; therefore, residual confounding cannot be excluded. This limitation should be considered when interpreting the comparative efficacy results. In addition, adherence and safety data were based on patient self-reports rather than objective measures such as pharmacy refill data, which may have introduced recall or reporting bias. Furthermore, the relatively short follow-up period limits the ability to assess whether improved adherence translates into long-term cardiovascular benefit, which remains the primary goal of lipid-lowering therapy in clinical practice.

## Conclusion

**Fig. 3:**
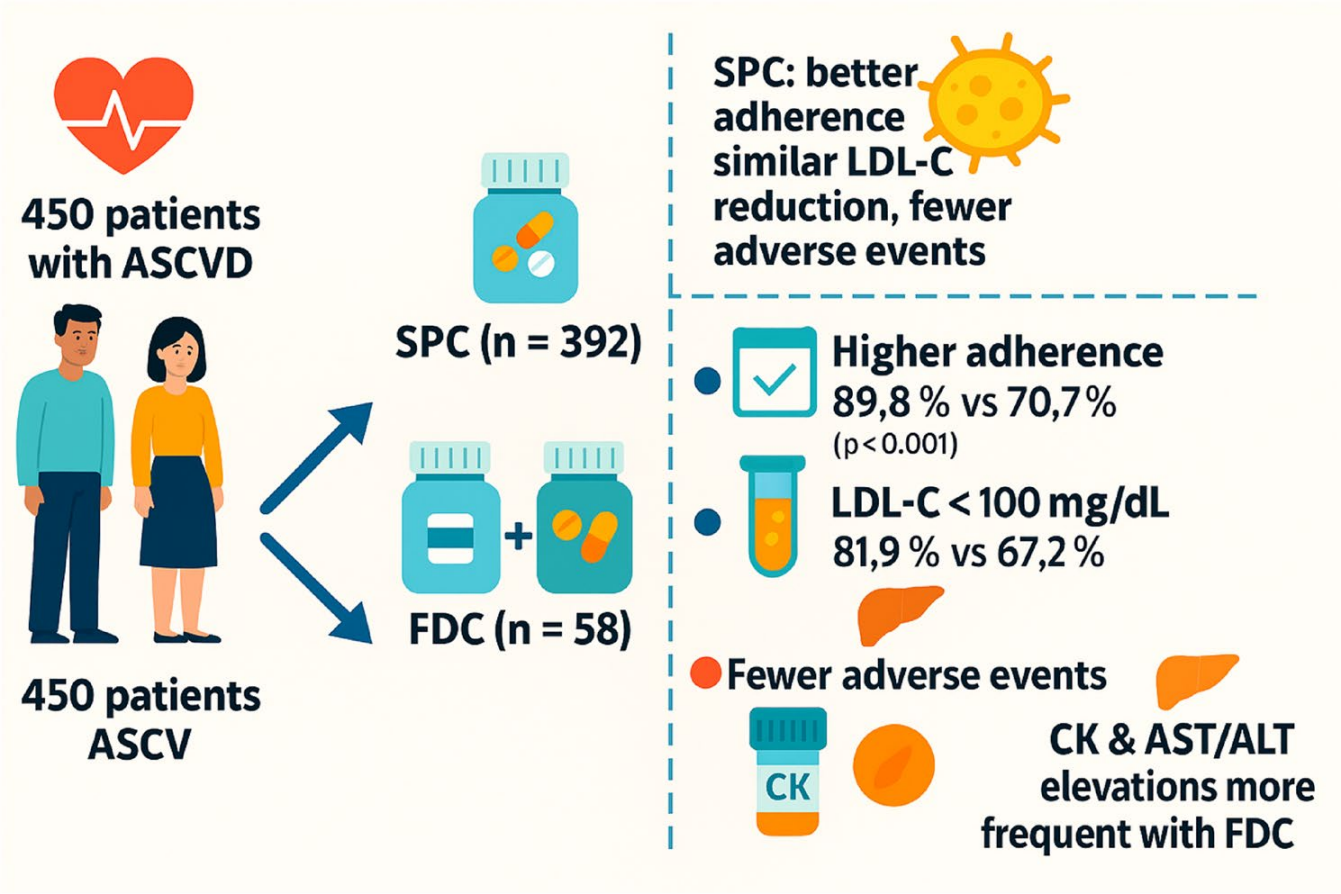
Central illustration summarizing study design, treatment regimens, and key outcomes comparing SPC and FDC strategies

In this real-world observational study, SPC therapy with atorvastatin and ezetimibe demonstrated significantly better treatment adherence compared to FDC, without compromising lipid-lowering efficacy or safety. Despite relatively lower statin doses, the SPC group achieved comparable LDL-C reductions and target attainment rates. These findings support the broader implementation of fixed-dose regimens as a practical strategy to enhance adherence and optimize CV risk management in patients with established ASCVD (Fig. 3).

## Data Availability

The datasets generated and/or analyzed during the current study are available from the corresponding author on reasonable request.
